# Modeling of a Broadband Microwave Composite Thin Film Absorber

**DOI:** 10.3390/mi14112119

**Published:** 2023-11-18

**Authors:** Ying Zhang, Yanze Gao, Suhui Yang, Zhuo Li, Xin Wang, Jinying Zhang

**Affiliations:** 1 School of Optics and Photonics, Beijing Institute of Technology, Beijing 100081, China; yzhang_phy@163.com (Y.Z.); suhuiyang@bit.edu.cn (S.Y.); lizhuo@bit.edu.cn (Z.L.); wangxin@bit.edu.cn (X.W.); jyzhang@bit.edu.cn (J.Z.); 2 Beijing Key Laboratory for Precision Optoelectronic Measurement Instrument and Technology, Beijing 100081, China

**Keywords:** thin absorber, broadband absorber, impedance matching, composite thin film

## Abstract

Composite thin film absorbers show superior performance and have a wide range of applications. Obtaining a broadband composite thin film absorber is a challenge. In this work, we proposed a modeling of a broadband microwave composite thin film absorber based on the impedance matching theory and equivalent circuit model of the square loop. The unit cell of the absorber was composed of metal square loops with high magnetic conductivity deposited on the polyethylene substrate, and an FR-4 (epoxy glass cloth) substrate was the spacer substrate layer. The simulation results show that the absorptivity of the absorber reached more than 90% in the frequency range of 8.7–18 GHz for TE and TM waves under normal incidence. The thickness of the designed absorber was 2.05 mm (0.059 λ_max_, λ_max_ corresponds to the maximum absorption wavelength). The simulation results show that the energy distribution in the proposed absorber was mainly localized in the top metal FSS layer due to the ohmic loss of metal, and the dielectric loss played a small role in the absorption of the absorber. Our work provides a design approach to improve the efficiency of optoelectronic devices and thermal detectors and has application prospects in radar and aircraft stealth applications.

## 1. Introduction

In recent years, composite film has developed rapidly and demonstrated its powerful ability to control electromagnetic waves. It exhibits superior properties that natural materials cannot possess, such as negative refraction [1], superlens effect [2], electromagnetic cloak [3], and so on. Composite film absorbers show superior performance and have a wide range of applications in thermal detectors [4,5] and cloaking technology [6]. Impedance matching between free space and the absorber is achieved by adjusting the effective permittivity and permeability to reduce reflection. The absorption of the electromagnetic waves depends on the ohmic loss and dielectric loss of the absorber. Landy et al. implemented a narrow-band absorber with a 96% absorptivity at 11.48 GHz [7]. The absorber consisted of a metal wire and an open resonant ring array. Although composite film absorbers have the advantages of thin thickness and good absorptivity, and the narrow-band absorber has found application prospects in some fields in recent years [8,9,10], broadband absorbers are more conducive to a wider range of practical applications. Therefore, many researchers have been working to increase the bandwidth of absorbers by, for example, creating absorbers with metal array structures of different sizes on the same surface [11,12,13,14], a multi-layer superposed pyramid-type or planar thin film with a stack structure [15,16,17], and so on. However, the manufacturing process of the multi-layer structure is complex. To ensure the positions of different layer structures, high levels of processing accuracy are required. In addition, the thickness of absorbers with a multi-layer structure is generally thick; this adds to the weight and cost of the stealth surface. Recently, conductive films have been proposed to design broadband absorbers, such as indium tin oxide (ITO) with surface resistance [18,19,20]. Zhang et al. introduced an ITO as the frequency selective surface (FSS) to form the top layer and reflective bottom plate. The optically transparent metamaterial absorber achieved broadband microwave absorption [19]. With ITO composite film, one can increase the broadband absorption and reduce the thickness of the absorber. However, for the larger stealth surface in practical application, the preparation of ITO film with a larger size currently cannot be achieved. So, obtaining a broadband composite thin film absorber is a challenge.

To solve this problem, we proposed a modeling of a broadband microwave composite thin film absorber based on the impedance matching theory [21] and equivalent circuit model of the square loop [22,23]. The unit cell of the absorber was composed of metal square loops with high magnetic conductivity deposited on the polyethylene (PET) substrate, and an FR-4 (epoxy glass cloth) substrate was the spacer substrate layer. The simulation results show that the absorptivity of the absorber reached more than 90% in the frequency range of 8.7–18 GHz for TE and TM waves under normal incidence. The average absorptivity in the frequency range of 8.7–18 GHz was 95.1%. The designed absorber was thinner, only 2.05 mm (0.059 λ_max_). The energy distribution in the proposed absorber was mainly localized in the top metal FSS layer due to the ohmic loss of metal, and the dielectric loss played a small role in the absorption of the absorber. This type of absorber has application prospects in radar and aircraft stealth applications.

## 2. Absorber Design and Methods

**Figure 1:**
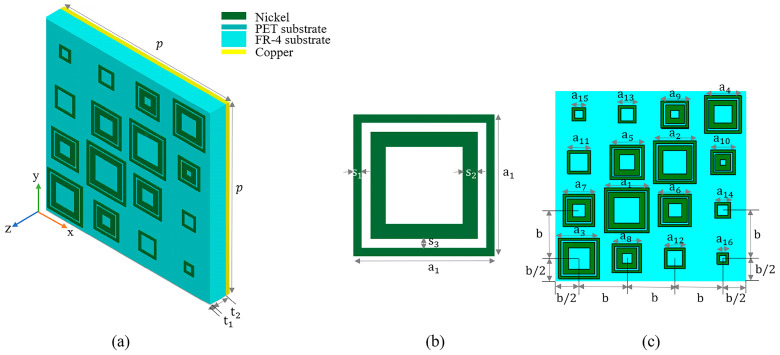
(**a**) Schematic perspective view of the unit cell of the designed microwave absorber. (**b**) Partial enlarged view. (**c**) Front view.

The structure of the unit cell of the designed microwave absorber is depicted in Figure 1. The nickel square loop array was used as an FSS to form the top layer. Nickel has an electric conductivity of $\sigma_{1}=1.44\times 10^{7}\left[S/m\right]$ and a magnetic conductivity of $\mu_{1}=600$. The thickness of the nickel layer was 0.018 mm and the sizes of the square loop were optimized as follows: The geometry parameters were designed as $a_{1}$ = 5.1 mm, $a_{2}$ = 4.8 mm, $a_{3}$ = 4.6 mm, $a_{4}$ = 4.2 mm, $a_{5}$ = 3.9 mm, $a_{6}$ = 3.7 mm, $a_{7}$ = 3.6 mm, $a_{8}$ = 3.3 mm, $a_{9}$ = 3.1 mm, $a_{10}$ = 2.8 mm, $a_{11}$ = 2.6 mm, $a_{12}$ = 2.3 mm, $a_{13}$= 2 mm, $a_{14}$ = 1.8 mm, $a_{15}$ = 1.5 mm, and $a_{16}$ = 1.3 mm, respectively. The substrate was polyethylene (PET); the PET had a relative permittivity of $\varepsilon_{1}=3.0\left(1-j0.06\right)$ and thickness of $t_{1}$ = 0.05 mm. The middle FR-4 substrate had relative permittivity of $\varepsilon_{2}=4.3\left(1-j0.025\right)$ and thickness of $t_{2}$ = 2 mm. The bottom layer was a uniform copper plate with an electric conductivity of $\sigma_{2}=5.8\times 10^{7}\left[S/m\right]$, and the thickness was 0.018 mm. The period length of the unit cell was *p*, *p* = 21.6 mm. The width of the nickel outer square loop $s_{1}$ was 0.3 mm, the width of the inner square loop $s_{2}$ was 0.6 mm, and the distance between the two square loops $s_{3}$ was 0.2 mm. The square loops were arranged equally in the center spacing with a spacing of b = 5.4 mm.

**Figure 2:**
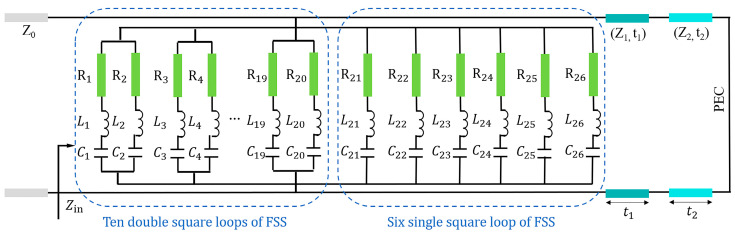
The equivalent circuit of the unit cell of the absorber.

Based on the impedance matching theory [21] and equivalent circuit model of the square loop [22,23], we conducted modeling of a microwave absorber. The equivalent circuit of the unit cell of the absorber is depicted in Figure 2. The unit cell contained several square loops. The single square loop was equivalent to a resistance-inductance-capacitance (R-L-C) circuit. The combined effect of all square loops was equivalent to connecting all square loops parallelly. The equivalent circuit is depicted in the dashed box in Figure 2. The resistance was generated by the ohm lossy property of FSS, inductance was generated by the square loop, and capacitance was generated by gaps between adjacent loops.

The double square loops were equivalent to two parallel resistance-inductance-capacitance (R-L-C) circuits. The $L_{n}$ *−* $C_{n}$ effective circuit was derived for the double square loop array. At normal incidence, the inductance $\left(L_{n}\right)$ and capacitance $\left(C_{n}\right)$ of the double square loops of FSS were calculated by the following formulas [22]. The equivalent circuit for the double square loop array was derived from reference [22]. The basic equations for calculating the inductance and capacitance of strip gratings are found in Marcuvitz.

(1) $$\frac{\omega L_{n1}}{Z_{0}}=2\frac{a_{n}}{p}\frac{F\left(p,s_{1},\lambda \right)F\left(p,s_{2},\lambda \right)}{F\left(p,s_{1},\lambda \right)+F\left(p,s_{2},\lambda \right)}$$

(2) $$\frac{\omega L_{n2}}{Z_{0}}=F\left(p,2s_{2},\lambda \right)\frac{a_{n}-2\left(s_{1}+s_{2}\right)}{p}$$

(3) $$\omega C_{n1}\cdot Z_{0}=3\frac{a_{n}}{p}F\left(p,p-a_{n},\lambda \right)$$

(4) $$\omega C_{n2}\cdot Z_{0}=4\frac{a_{n}-2\left(s_{1}+s_{2}\right)}{p}\frac{F\left(p,p-a_{n},\lambda \right)F\left(p,s_{3},\lambda \right)}{F\left(p,p-a_{n},\lambda \right)+F\left(p,s_{3},\lambda \right)}$$

(5) $$F\left(p,s,\lambda \right)=\frac{p}{\lambda }\left[\ln\left(\csc\frac{\pi s}{2p}\right)+G\left(p,s,\lambda \right)\right]$$

(6) $$G\left(p,s,\lambda \right)=\frac{{\left(1-\beta^{2}\right)}^{2}\left[A(1-\frac{\beta^{2}}{4})+2\beta^{2}A^{2}\right]}{\left(1-\frac{\beta^{2}}{4}\right)+2A\beta^{2}\left(1+\frac{\beta^{2}}{2}-\frac{\beta^{4}}{8}\right)+2A^{2}\beta^{6}}$$

(7) $$A=\frac{1}{\sqrt{1-{\left(\frac{p}{\lambda }\right)}^{2}}}-1$$

(8) $$\beta =\sin\left(\frac{\pi s}{2p}\right)$$

where $L_{n1}$*,* $L_{n2}$, $C_{n1}$, and $C_{n2}$ are the inductance and capacitance of the double square loops, respectively. *ω* is the operating radian frequency and λ is the wavelength. $Z_{0}$ is the impedance of the air. *p* is the period of the unit cell, $a_{n}$ is the side length of the square loop, $s_{1}$ is the outer square loop width, $s_{2}$ is the inner square loop width, and $s_{3}$ is the distance between the two square loops. *s* in (5), (6), and (8) equals $s_{1}$, $s_{2}$, $2s_{2}$, *p −* $a_{n}$, and $s_{3}$ to calculate $L_{n1}$*,* $L_{n2}$, $C_{n1}$, and $C_{n2}$.

The $L_{N}$*-*$C_{N}$ effective circuit was derived for the single square loop array. At normal incidence, the inductance ($L_{N}$) and capacitance ($C_{N}$) of the single square loop of FSS were calculated by the following formulas [23]. The inductance and capacitance were determined by the parameters of a single square loop of FSS.

(9) $$\frac{\omega L_{N}}{Z_{0}}=\frac{a_{N}}{p}F\left(p,2s_{1},\lambda \right)$$

(10) $$\omega C_{N}\cdot Z_{0}=4\frac{a_{N}}{p}F\left(p,p-a_{N},\lambda \right)$$

where $L_{N}$ and $C_{N}$ are the inductance and capacitance of the single square loop, respectively. *ω* is the operating radian frequency and λ is the wavelength. $Z_{0}$ is the impedance of the air. *p* is the period of the unit cell, $a_{N}$ is the side length of the square loop, and $s_{1}$ is the loop width. *s* in (5), (6), and (8) equals *2*$s_{1}$ and *p-*$a_{N}$ to calculate $L_{N}$ and $C_{N}$.

The ohm resistance of FSS was determined as

(11) $$R_{i}=\frac{1}{\sigma \sqrt{2/\omega \mu \sigma }}$$

where *ω* is the operating radian frequency, *µ* is the effective permeability of the metal square loop, and *σ* is the effective conductivity of the metal square loop. The combined effect of all square loops was equivalent to connecting all square loops parallelly. The total impedance of FSS was defined as follows [24]:

(12) $$Z_{FSS}=R+j\left(\omega L-\frac{1}{\omega C}\right)$$

where R is the total effective resistance of FSS, L is the total effective inductance of FSS, and *C* is the total effective capacitance of FSS.

The two-port network model could be used to calculate the reflection of the cascading structure by multiplying ABCD matrices in series [25,26]. The ABCD matrix of a dielectric layer under the normal incidence is defined as

(13) $$\left[\begin{array}{cc} A_{i} & B_{i}\\ C_{i} & D_{i} \end{array}\right]=\left[\begin{array}{cc} \cos h\left(k_{i}t_{i}\right) & jZ_{i}\sin h\left(k_{i}t_{i}\right)\\ \frac{j}{Z_{i}}\sin h\left(k_{i}t_{i}\right) & \cos h\left(k_{i}t_{i}\right) \end{array}\right]$$

where $k_{i}=\frac{2\pi }{\lambda }\sqrt{\varepsilon_{i}}$ and $Z_{i}=Z_{0}\frac{1}{\sqrt{\varepsilon_{i}}}$ are the wavenumber and intrinsic impedance of the dielectric layer (PET and FR-4 substrate), respectively. $t_{i}$ and $\varepsilon_{i}$ are the thickness and complex permittivity of the dielectric layer (PET and FR-4 substrate), respectively. The ABCD matrix of the nickel FSS was defined as

(14) $$\left[\begin{array}{cc} A_{FSS} & B_{FSS}\\ C_{FSS} & D_{FSS} \end{array}\right]=\left[\begin{array}{cc} 1 & 0\\ \frac{1}{Z_{FSS}} & 1 \end{array}\right]$$

The proposed equivalent circuit model can be regarded as a two-port network being short-circuited at the end. By generating the transmission matrix of each segment, the total transfer ABCD matrix could be obtained. The total transfer ABCD matrix of the composite film is defined as

(15) $$\left[\begin{array}{cc} A & B\\ C & D \end{array}\right]=\left[\begin{array}{cc} A_{FSS} & B_{FSS}\\ C_{FSS} & D_{FSS} \end{array}\right]\left[\begin{array}{cc} A_{1} & B_{1}\\ C_{1} & D_{1} \end{array}\right]\left[\begin{array}{cc} A_{2} & B_{2}\\ C_{2} & D_{2} \end{array}\right]$$

where the coefficients *A*, *B*, *C*, and *D* represent the terms of the total transfer ABCD matrix of the composite film. The composite film was composed of a frequency selective surface (FSS) and a general number of dielectrics.

The reflection coefficient of the absorber was defined as

(16) $$\Gamma =\frac{A+B/Z_{0}-CZ_{0}-D}{A+B/Z_{0}+CZ_{0}+D}$$

The reflectivity was $R={\left|\Gamma \right|}^{2}$.

Based on the impendence matching theory [21], the reflection coefficient of the absorber was defined as

(17) $$Z_{i}=jZ_{0}\tan\left(k_{i}t_{i}\right)/\sqrt{\varepsilon_{i}}$$

(18) $$Z_{in}=\frac{Z_{FSS}Z_{1}Z_{2}}{Z_{Fss}\left(Z_{1}+Z_{2}\right)+Z_{1}Z_{2}}$$

(19) $$\Gamma =\frac{Z_{in}-Z_{0}}{Z_{in}+Z_{0}}$$

The reflectivity was $R={\left|\Gamma \right|}^{2}$. For details about the physical parameters, see the previous description. According to (19), if the impedance of the absorber $Z_{in}$ matches the impedance of the air $Z_{0}$,

(20) $$Z_{in}=Z_{0}$$

The reflectivity was zero because the bottom layer of the composite thin film absorber was copper with a thickness of 0.018 mm, which was much thicker than the skin depth of the incident wave in this frequency band, so the transmittance was zero. Thus, the absorptivity was *A* = 1 − *R*.

We performed simulations of the absorption properties of the absorber using CST (computer simulation technology) Microwave Studio [27]. For the electromagnetic field full-wave simulation, the finite integration technique (FIT) was used. Full wave simulation refers to solving the unapproximated Maxwell’s equations in boundary conditions in the simulation. Periodic boundary conditions were adopted in the x and y directions. The distribution of ε and μ was a periodic function in the x, y direction, with period *p* satisfying ε(x + *p*) = ε(x), μ(x + *p*) = μ(x), ε(y + *p*) = ε(y), and μ(y + *p*) = μ(y); an open boundary condition was adopted in the z direction. The structure of the unit cell for the absorber is depicted in Figure 1. The working frequency ranged from 8 GHz to 18 GHz.

**Figure 3:**
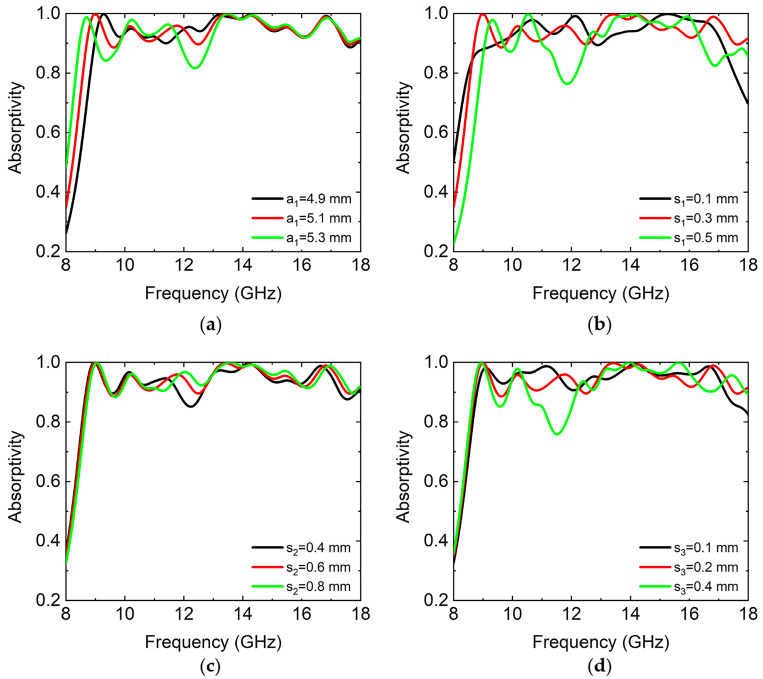
The simulated absorption spectrums of the absorber with different side lengths of the square loop (**a**), outer square loop widths (**b**), inner square loop widths (**c**), and distances between the two square loops (**d**) at normal incidence of TE wave, respectively. $a_{1}$ = 4.9, 5.1, and 5.3 mm; $s_{1}$ = 0.1, 0.3, and 0.5 mm; $s_{2}$ = 0.4, 0.6, and 0.8 mm; and $s_{3}$ = 0.1, 0.2, and 0.4 mm, respectively.

In order to expand the absorption bandwidth, we optimized the side lengths of the square loops on the top metal FSS of the absorber. The side length of the square loop decreased in turn. For this, we also nested a square loop inside a square loop. The absorption spectrums of the absorber with different side lengths of the square loop ($a_{1}$), outer square loop widths ($s_{1})$, inner square loop widths ($s_{2})$, and distances between the two square loops ($s_{3})$ were simulated at normal incidence of the TE wave, as shown in Figure 3, respectively. We set the side lengths of the square loop as $a_{1}$ = 4.9, 5.1, and 5.3 mm, respectively. From Figure 3a, we found that when $a_{1}$ increased, the absorption spectrum was redshifted and enhanced low-frequency absorption. But, when $a_{1}$ = 5.3 mm, the local absorption intensity decreased. The average absorptivity in the frequency range of 8.7–18 GHz was 95.1%, 95.1%, and 94.4%, respectively. We set the outer square loop widths as $s_{1}$ = 0.1, 0.3, and 0.5 mm, respectively. From Figure 3b, it can be seen that when $s_{1}$ increased, the absorption bandwidth increased at first and then decreased. When $s_{1}$ = 0.1 mm, the local absorption intensity decreased in the frequency range of 17–18 GHz. When $s_{1}$ = 0.5 mm, the local absorption intensity decreased in the frequency range of 11–12.5 GHz. The average absorptivity in the frequency range of 8.7–18 GHz was 94.3%, 95.1%, and 92.6%, respectively. We set the inner square loop widths as $s_{2}$ = 0.4, 0.6, and 0.8 mm, respectively. From Figure 3c, we found that when $s_{2}$ increased, the change in the absorption spectrum was not obvious. The local absorption intensity increased in the frequency range of 11.7–12.7 GHz. The average absorptivity in the frequency range of 8.7–18 GHz was 94.4%, 95.1%, and 95.5%, respectively. We set the distances between the two square loops as $s_{3}$ = 0.1, 0.2, and 0.4 mm, respectively. From Figure 3d, we found that when $s_{3}$ increased, the absorption bandwidth increased at first and then decreased. The local absorption intensity decreased. The average absorptivity in the frequency range of 8.7–18 GHz was 95.4%, 95.1%, and 93.9%, respectively. Based on the influence of the geometric parameters of the absorber on the absorption performance, we optimized the absorption performance of the absorber. In the process of parameter optimization, we comprehensively considered the average absorptivity and absorption bandwidth to select the best parameter value. The optimized geometric parameters of the absorber are described in Figure 1.

**Figure 4:**
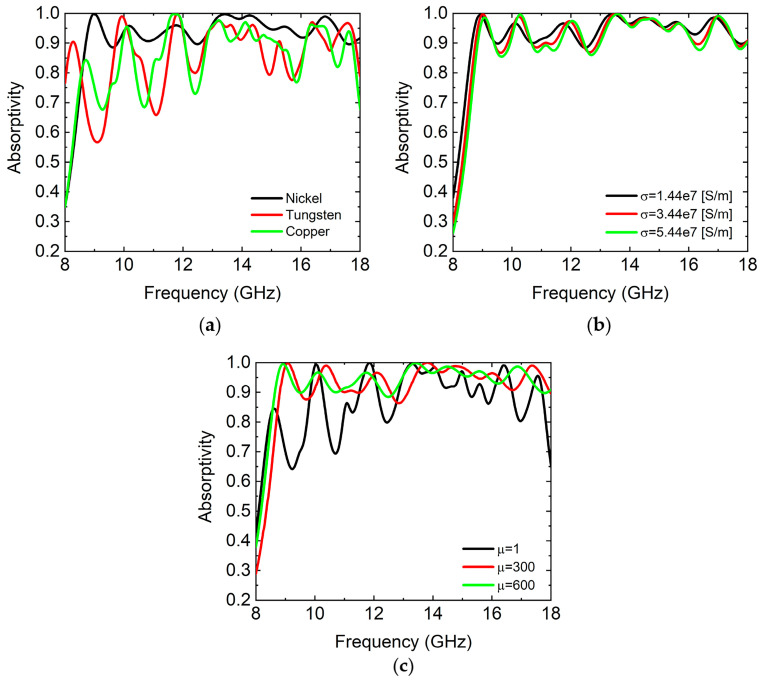
At normal incidence of the TE wave, the simulated absorption spectrums of the absorber in the case of the top metal FSS layer with different electrical conductivities and magnetic conductivities. (**a**) The metal FSS of the top layer was nickel, tungsten, and copper, respectively. The simulated absorption spectrums of the absorber in the case of the top metal FSS layer with different electrical conductivities (**b**) and magnetic conductivities (**c**).

We studied the influence of the material properties of the top metal FSS on the absorption properties of the absorber. Under normal incidence of the TE wave and TM wave, the metal FSSs (square loops) of the top layer with different electrical conductivities and magnetic conductivities were considered in the simulations, as shown in Figure 4. Other parameters of each component of the absorber are described in Figure 1. Here, we chose nickel, tungsten, and copper as metal FSSs on the top layer, respectively. The electrical conductivity of nickel, tungsten, and copper are $\sigma_{Nickel}=1.44\times 10^{7}[S/m$], $\;\sigma_{Tungsten}=1.89\times 10^{7}[S/m$], and $\;\sigma_{Copper}=5.8\times 10^{7}[S/m$], respectively, and the magnetic conductivity of nickel, tungsten, and copper are $\mu_{Nickel}=600$, $\;\mu_{Tungsten}=1$, and $\;\mu_{Copper}=1$. The absorption spectrums are shown in Figure 4a. The average absorptivity in the frequency range of 8.7–18 GHz was 95.1%, 88%, and 88.3%, respectively. We can see that the average absorptivity and absorption bandwidth of the absorber in the case of the top metal FSS formed by nickel were the highest and widest. Nickel has low electrical conductivity and high magnetic conductivity. In addition, we studied the contribution of electrical conductivity and magnetic conductivity to the absorption of the absorber, respectively. When μ=600, $\sigma =1.44\times 10^{7},\;3.44\times 10^{7},\;\mathrm{and}\;5.44\times 10^{7}\left[S/m\right]$, respectively. The simulated absorption spectrums of the absorber in the case of the top metal FSS layer with different electrical conductivities are shown in Figure 4b. The average absorptivity in the frequency range of 8.7-18 GHz was 95.1%, 94.6%, and 94%, respectively. We can see that the average absorptivity and absorption bandwidth decreased with the increase in the electrical conductivity. When $\sigma =1.44\times 10^{7}\left[S/m\right]$, μ=1, 300, and 600, respectively. The simulated absorption spectrums of the absorber in the case of the top metal FSS layer with different magnetic conductivities are shown in Figure 4c. The average absorptivity in the frequency range of 8.7–18 GHz was 89.7%, 94.6%, and 95.1%, respectively. We can see that the average absorptivity and absorption bandwidth increased with the increase in the magnetic conductivity. Figure 4 shows that the contribution of the high magnetic conductivity of nickel FSS to the absorption of the absorber was dominant. It was indicated that the absorber with high magnetic conductivity had higher absorptivity than the ones with low magnetic conductivity. By studying the effects of different metal materials of the top metal FSS layer on the absorption properties of the absorber, we find that the absorber in the case of the top metal FSS layer with low electrical conductivity and high magnetic conductivity had a better absorption effect. Therefore, when designing the absorber, a metal FSS with low electrical conductivity and high magnetic conductivity was selected.

**Figure 5:**
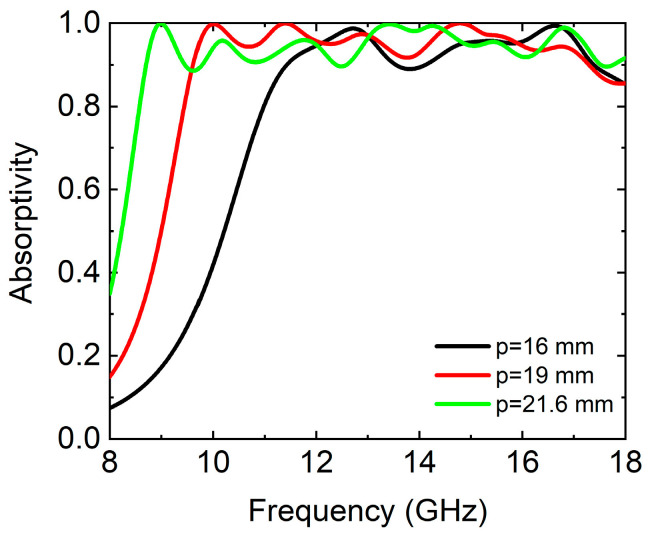
The simulated absorption spectrums of the absorber with different period lengths at normal incidence of TE wave, respectively. *p* = 16, 19, and 21.6 mm, respectively.

We scaled the absorber to equal proportions and set the period lengths as *p* = 16, 19, and 21.6 mm, respectively. The absorption spectrums of the absorber with different period lengths were simulated at normal incidence of the TE wave, as shown in Figure 5, respectively. From Figure 5, we find that when *p* increased, the absorption spectrum was redshifted and enhanced low-frequency absorption. The average absorptivity in the frequency range of 8.7–18 GHz was 86.3%, 94.1%, and 95.1%, respectively.

As mentioned above, we optimized the parameters and materials of the absorber. In the process of parameter optimization, we comprehensively considered the average absorptivity and absorption bandwidth to select the optimal values. Natural materials close to optimal values were selected. The metal FSS of the top layer was nickel, tungsten, and copper, respectively. By studying the effects of different metal materials of the top metal FSS layer on the absorption properties of the proposed absorber, we found that the absorber in the case of the top metal FSS layer with low electrical conductivity and high magnetic conductivity had a better absorption effect. Therefore, we chose nickel as the metal FSS of the absorber. In addition, in order to expand the absorption bandwidth, we optimized the side lengths of the square loops on the top metal FSS of the absorber. The side length of the square loop decreased in turn. For this, we also nested a square loop inside a square loop. With this method, the optimized geometric parameters and materials of the absorber were obtained, as shown in Figure 1.

**Figure 6:**
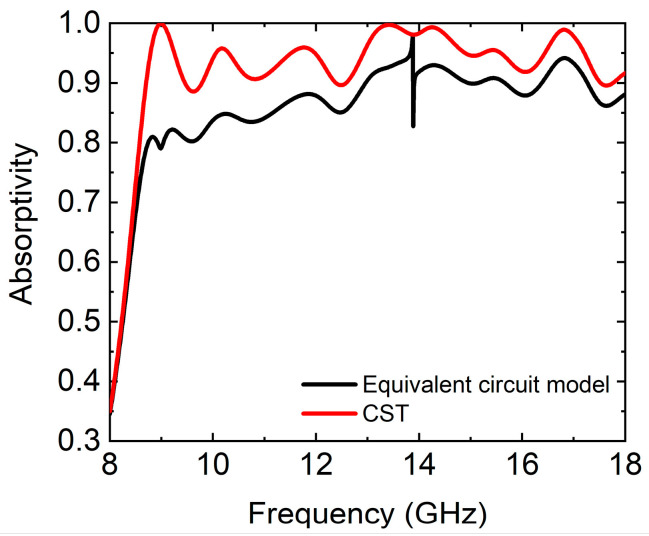
The simulated absorption spectrums of the absorber with equivalent circuit model and software CST, respectively.

The equivalent circuit model and software CST 2019 were used to simulate the absorption spectrums of the absorber at normal incidence of the TE wave, respectively. The simulation results are shown in Figure 6. The average absorptivity in the frequency range of 8.7–18 GHz was 95.1% and 87.8%, respectively. The two simulation results were in good agreement.

## 3. Results and Discussion

**Figure 7:**
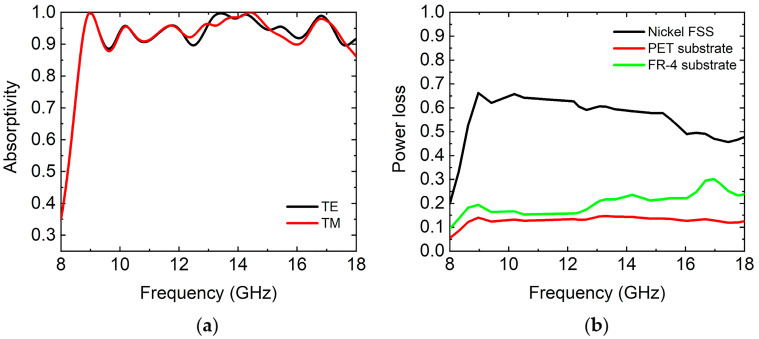
(**a**) The simulated absorption spectrums of the absorber at normal incidence of TE and TM waves. (**b**) The simulated power loss of each component of the proposed absorber.

We simulated the absorption spectrums of the thin film absorber at normal incidence of the TE and TM waves, as shown in Figure 7a. The simulated results show that the absorptivity of the absorber reached more than 90% in the frequency range of 8.7–18 GHz for the TE wave and near 100% perfect absorption at some resonant frequencies. As for the TM wave, the absorptivity of the absorber reached more than 90% in the frequency range of 8.7–17.65 GHz. The average absorptivity in the frequency range of 8.7–18 GHz for the TE and TM waves was 95.1% and 95%, respectively. Then, we simulated the power loss of each component of the proposed absorber, as shown in Figure 7b. From the simulation results, the nickel FSS contributed to the major power loss in the absorber. Moreover, the PET substrate and FR-4 substrate also contributed to the partial power loss. Thus, three components played a joint role in the absorption of the designed thin broadband microwave absorber. The energy distribution in the proposed absorber was mainly confined to the nickel FSS due to the ohmic loss of the nickel FSS, and the dielectric loss played a small role in the absorption of the absorber.

**Figure 8:**
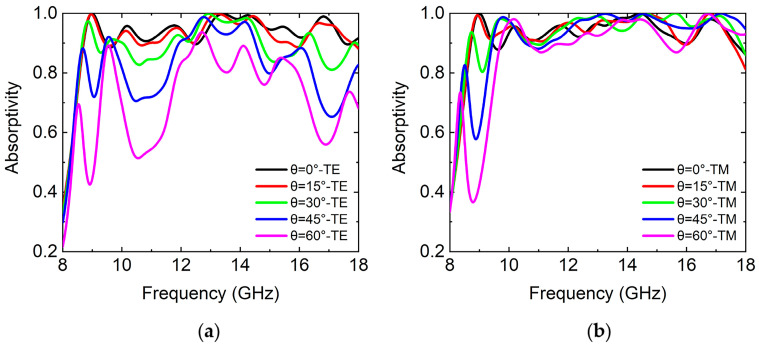
The simulated absorption spectrums of the absorber under oblique incidence. (**a**) TE wave and (**b**) TM wave with an incident angle of 0°, 15°, 30°, 45°, and 60°, respectively.

We simulated the absorption properties of the absorber with incident angles from 0° to 60°, as shown in Figure 8, respectively. The simulation results show that the variation of the absorptivity of the absorber for the TE wave was more obvious than that of the TM wave. Under oblique incidence of the TE wave, as the incident angle increased, the absorptivity of the absorber decreased. At the incident angle of 30°, the absorptivity dropped to 82.2%. When the incident angle increased to 45°, the absorptivity dropped to 65%. But, in the frequency range of 11.9–14.5 GHz, the absorptivity of the absorber was more than 90%. When the incident angle increased to 60°, the absorptivity of the absorber decreased significantly. When the incident angles ranged from 0° to 60° for the TE wave, the average absorptivity in the frequency range of 8.7–18 GHz was 95%, 94%, 91%, 85%, and 75%, respectively. Under oblique incidence of the TM wave, as the incident angle increased to 60°, the absorptivity of the absorber could be maintained above 87% in a wide range of frequency bands. When the incident angles ranged from 0° to 60° for the TM wave, the average absorptivity in the frequency range of 8.7–18 GHz was 95%, 95%, 96%, 96%, and 92%, respectively. This was due to the fact that the orientation of the magnetic field was maintained when the incident angle was varied. It could effectively keep the strength of magnetic resonance at all of the incident angles. The difference in the absorptivity for the TE and TM waves lay in the characteristic impedance of each layer of the absorber, expressed as $Z_{i}sec\theta_{i}$ and $Z_{i}cos\theta_{i}$, respectively, where $Z_{i}=\sqrt{\mu_{i}/\varepsilon_{i}}$ is the characteristic impedance of the material and $\theta_{i}$ is the incident angle.

**Figure 9:**
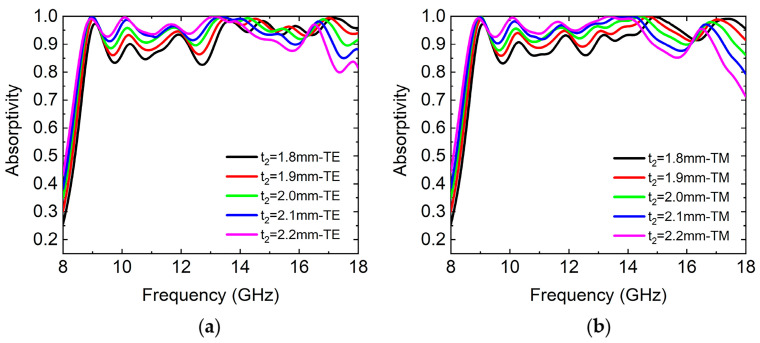
The simulated absorption spectrums in the case of FR-4 substrates with different thicknesses. (**a**) TE wave and (**b**) TM wave. $t_{2}$ = 1.8, 1.9, 2, 2.1, and 2.2 mm, respectively.

The thickness of the absorber was also a key factor in the design of the absorber. We simulated the influence of the thickness of the FR-4 substrate on the absorption properties of the absorber, as shown in Figure 9, respectively. Based on the optimized parameters of the absorber, we set the thickness as $t_{2}$ = 1.8, 1.9, 2, 2.1, and 2.2 mm, respectively. It can be seen that when the thickness $t_{2}$ of the FR-4 substrate increased, the absorptivity of the absorber increased in a certain band, but the absorption bandwidth increased at first and then decreased. When the thickness $t_{2}$ of the FR-4 substrate increased for the TE wave, the average absorptivity in the frequency range of 8.7–18 GHz was 93.2%, 94.5%, 95.1%, 95.1%, and 94.5%, respectively. When the thickness $t_{2}$ of the FR-4 substrate increased for the TM wave, the average absorptivity in the frequency range of 8.7–18 GHz was 93.2%, 94.3%, 94.9%, 94.8%, and 94.5%, respectively. It can be seen that when $t_{2}$ = 2 mm, the average absorptivity of the absorber was at the maximum. Taking into account average absorptivity and absorption bandwidth, the best value of $t_{2}$ for the design was 2 mm. We found that when $t_{2}$ increased, the absorption spectrum was redshifted. This can be explained by the path phase $\varphi_{Pl}$ of the incident electromagnetic wave propagating in the dielectric layer, expressed by Formula (21):

(21) $$\varphi_{Pl}=\frac{4t_{2}\sqrt{\varepsilon_{2}-\sin^{2}\theta }}{\lambda }$$

where $t_{2}$ is the thickness of the FR-4 substrate, *λ* is the wavelength of the electromagnetic wave, $\varepsilon_{2}$ is the real part of the permittivity of the FR-4 substrate, and *θ* is the incident angle of the electromagnetic wave. Assuming that the plane wave is in normal incidence, $\varphi_{Pl}$ and $\varepsilon_{2}$ are a certain value and sinθ=0, so that the thickness $t_{2}$ of the dielectric is inversely proportional to the absorption spectral frequency.

## 4. Conclusions

In this work, a modeling of a broadband microwave composite thin film absorber was proposed. The proposed absorber consisted of a copper backboard, an epoxy glass cloth substrate spacer layer, and a nickel FSS deposited on the polyethylene substrate. Nickel with high magnetic conductivity in a nested square loop array was used as the top layer of the absorber. We studied the influences of the geometric parameters and material properties of the top metal FSS layer, incident angles, and thickness of the absorber on the absorption properties of the absorber. Moreover, we simulated the power loss of each component of the proposed absorber. The energy distribution in the proposed absorber was mainly localized in the top metal FSS layer due to the ohmic loss of the nickel FSS. The simulation results show that at normal incidence of TE and TM waves, the absorptivity of the absorber reached more than 90% in the frequency range of 8.7–18 GHz. The average absorptivity in the frequency range of 8.7–18 GHz was 95.1%. In addition, the average absorptivity of the absorber could be maintained above 75% and 92% under oblique incident angles from 0° to 60° of TE and TM waves, respectively. Moreover, the designed absorber had a thin thickness; the thickness was 2.05 mm (0.059 λ_max_). By studying the influence of the material properties of the top metal FSS layer on the absorption properties of the absorber, we found that the absorber in the case of the top metal FSS layer with low electrical conductivity and high magnetic conductivity had a better absorption effect. Therefore, when designing and fabricating absorbers, a metal FSS with low electrical conductivity and high magnetic conductivity should be selected. Finally, the proposed design method of the absorber can help improve the efficiency of photoelectric devices and thermal detectors and has application prospects in microelectromechanical systems and photodetector, radar, and aircraft stealth.

## Data Availability

Data available on request due to restrictions eg privacy. The data presented in this study are available on request from the corresponding author. The data are not publicly available due to privacy.
